# Tire-Derived Organic Chemicals in Urban Air at the Source-Sector Scale and Guidance on the Application of Polyurethane Foam Disk Passive Air Samplers

**DOI:** 10.1021/acsestair.5c00013

**Published:** 2025-03-28

**Authors:** Cassandra Johannessen, Amandeep Saini, Xianming Zhang, Tom Harner

**Affiliations:** † Department of Chemistry and Biochemistry, Concordia University, Montreal, Quebec H4B 1R6, Canada; ‡ Air Quality Processes Research Section, Environment & Climate Change Canada, Toronto, Ontario M3H 5T4, Canada

**Keywords:** urban air pollution, 6PPD-quinone, antioxidants, transformation products, passive air sampling, PUF disk

## Abstract

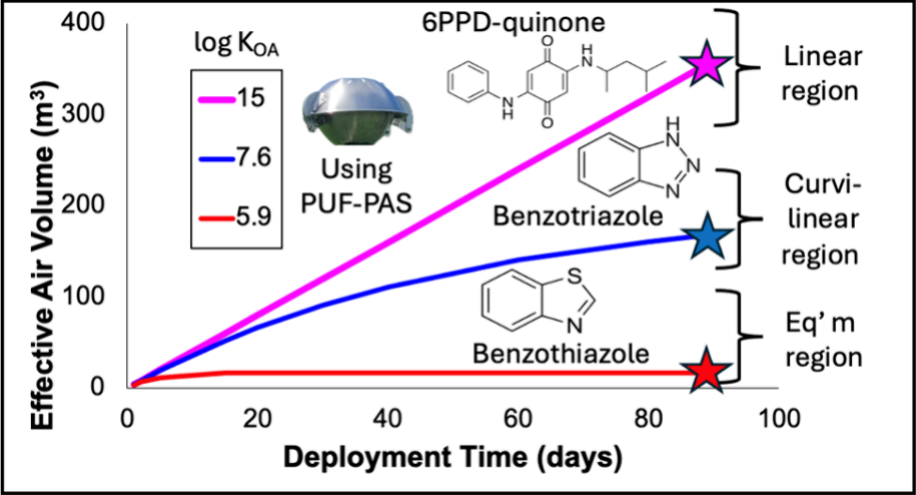

Tire-derived chemicals (TDCs) are shown to be elevated in urban environments. In this study, we analyzed 6PPD-quinone, 2,2,4-trimethyl-1,2-dihydroquinoline (TMQ), hexa(methoxy)methylmelamine (HMMM), as well as selected benzothiazoles and benzotriazoles, in different urban source-sectors. The chemical analyses were conducted on archived extracts of polyurethane foam (PUF) disk passive air samplers deployed across eight locations (including residential, industrial, semiurban, and traffic areas) over successive 2-month periods in the Greater Toronto Area, Canada. Principal component analysis showed distinct profiles in traffic-heavy locations, where benzothiazole and 6PPD-quinone had maximal concentrations of 2100 pg/m^3^ and 3.4 pg/m^3^, and where several TDCs including 6PPD-quinone, benzotriazoles, and some benzothiazoles were elevated during winter months. HMMM had elevated concentrations in nontraffic sectors, suggesting anthropogenic sources other than tires. This study recognizes the unique challenges to accurately quantifying TDCs in ambient air and that results presented here should be considered semiquantitative. To reduce uncertainty, temperature-dependent PUF disk-air partition coefficients (K_PUF-AIR_) and gas-particle partitioning fractions of TDCs in ambient air are presented. These are calculated from K_OA_ values derived from quantum chemical methods using COSMOtherm and show that TDCs span a wide range of volatilities and gas-particle partitioning behavior, with implications for atmospheric fate and exposure. Lastly, guidance is provided on future measures to evaluate and minimize degradation losses of TDCs during sampling, extraction, and storage.

## Abstract 2

Partitioning of tire-derived chemicals to PUF disk and ambient particles is predicted and used to improve measurements in urban air across different source areas. Guidance is provided for future studies.

## 1 Introduction

Urban centers are unique sources of air pollution.^1^ Because urban land use tends to be mixed (e.g., commercial, industrial, vehicular, residential), urban air contains a wide range of contaminants. The elevated pollution levels of urban environments have been associated with a variety of negative health outcomes, such as cognitive, developmental, and reproductive issues, cancer, respiratory and cardiovascular illness, and premature death.^2−5^

Traffic is one of the most prominent sources of air pollution in urban environments.^2,6^ Historically, much attention has been paid to the pollution produced by the combustion of fuels and tailpipe emissions. With improving vehicle emission standards, the relative contribution of tailpipe exhaust emissions to air pollution has been diminishing.^7,8^ However, high-traffic areas continue to be a significant contributor to air pollution due to emissions from nontailpipe sources, such as from car tires.^8^ As tire-wear particles (TWPs) are continually generated from abrasion between the tire and road, urban centers are hotspots for this pollution type due to their high-traffic densities and abundant roadways.^9−11^ TWPs have been shown to readily release tire-derived chemicals (TDCs) which can migrate to water or sorb to soil.^12−16^ Recent studies have also confirmed that high levels of compounds typically associated with tire contamination are present in urban air.^13,17−20^ With the extent of urbanization only set to increase in the foreseeable future, investigation into TDCs becomes crucial to protect urban environments and their inhabitants. Many TDCs are considered contaminants of emerging concern, and their occurrence and distribution in urban air remain poorly understood.

Due to their established link to tire and vehicle pollution, 17 contaminants were initially examined in this study, including: diphenylguanidine (DPG), diphenylamine, hexa(methoxymethyl)melamine (HMMM), and 2,2,4-trimethyl-1,2-dihydroquinoline (TMQ). Selected compounds from larger chemical classes that have been strongly associated with vehicle pollution and tire-wear, such as *p*-phenylenediamines (PPDs), benzothiazoles, and benzotriazoles, were also investigated. Many of these compounds have associated toxicity, which heightens their risk as air pollutants. Physicochemical properties and relevant monitoring and toxicity studies were previously highlighted by Johannessen et al. for select TDCs.^21^

Although these compounds have been associated with tire-wear and vehicle pollution, they have other anthropogenic uses that complicate their source attribution. HMMM is used primarily as a cross-linker in resins. It is found in adhesives, food packaging, tires, paints, and other coatings used on cans and automobiles, for example.^22^ It is also used as a marine paint and is suspected to be a component of aircraft coatings.^23−25^ TMQ is a chemical used in rubber and plastic products including tires, hoses, adhesive tapes, cables, and electrical wire, and is reported to be the most common antioxidant used in the rubber industry by volume.^26,27^ Often, TMQ is used in conjunction with 6PPD (N-(1,3-Dimethylbutyl)-N′-phenyl-p-phenylenediamine) and protective wax to create a complementary protection system.^28^ PPDs (phenyl-p-phenylenediamines), such as 6PPD, are used as antiozonants in tires and other rubber products due to their ability to readily oxidize.^26,27,29,30^ Indeed, multiple PPDs have been shown to oxidize into PPD-quinones in the environment whose environmental fate and occurrence are largely unknown.^13,19,31,32^ Benzotriazole, benzothiazole, and their respective derivatives (referred to collectively as benzotriazoles and benzothiazoles) are extremely versatile and can be found in a plethora of products ranging from antifreezes and antifogging agents to hydraulic fluids and tires.^27,33−36^ They also have a long history of use as corrosion inhibitors.^33,37,38^ In rubber products like tires, benzothiazoles typically act as vulcanization accelerators.^27^

The primary objective of this work was to gain insight into the occurrence and behavior of these compounds within a large urban area, as well as to identify and quantify the contribution of different urban sectors to their overall pollution levels. We report here the air concentrations of these compounds in passive air samples acquired from various locations within the Greater Toronto Area (GTA), the largest metropolitan area in Canada. This study utilizes archived sample extracts from a 2016–2017 source-sector scale study that were originally used to associate the contamination of flame retardants and polycyclic aromatic compounds (PACs, through codeployed samplers) with different source-sectors in the urban environment.^39,40^ The land use areas that were investigated included residential, industrial, semiurban, and traffic, as well as a park location that is near an airport. The chemical profile of each analyte was interpreted based on its presence, spatial distribution, and observed seasonal differences in each source-sector, which provides insight into its emission pattern and environmental behavior. Overall, these data can be used to inform future monitoring campaigns and targeted efforts for air pollution management.

A secondary objective of this work is to highlight sources of uncertainty in air measurements of TDCs and to provide tools and guidance for future work. The GAPS Template,^41^ a tool used to derive air concentrations for chemicals (gas+particle-phase) measured using polyurethane foam (PUF) disk type passive air samplers, is updated to include TDCs. This is made possible by new estimates of the octanol-air partition ratio, K_OA_, made using the quantum chemical COSMOtherm model.^42^ The new K_OA_ values for TDCs allow PUF disk-air partition coefficients (K_PUF-AIR_)^43^ to be derived as a function of temperature, which are then used to derive the equivalent air volume (V_eq_) for TDCs as well as their predicted particle-phase fractions using a K_OA_-based model.^44^ This improvement to the method is especially relevant for the more volatile TDCs with lower K_OA_ values, which are likely to approach equilibrium with PUF disks during sampler deployments.

## 2 Materials and Methods

### 2.1 Sampling and Sample Extraction

Polyurethane foam disk passive air samplers (PUF-PAS) (Tisch Environmental) were deployed at eight sampling sites across the GTA in Ontario Canada, as previously described by Saini et al.^39^ These samplers work to reflect the whole air mixture by sampling both particles and gas-phase components.^45^ PUF-PAS were deployed at each location for periods of ∼ 2 months. The initial sampling campaign consisted of 6 periods in total and spanned from August 2016 to September 2017. For the present study, archived sample extracts from Periods 2 to 6 (October 2016 – September 2017) were obtained. The eight study locations were at: Hanlan’s Point (PkAir-HP), Wallberg Building (TrafUrb-WB), Ontario Ministry of the Environment, Conservation and Parks (Traf-MECP), Downsview (SemiUrb-DV), Burlington (Ind-BUR), North York (Res-NY), Kennedy (Res-KE), and North Toronto (Res-NT). Each site was selected to represent distinct source-sectors (Figure S1), and detailed land use data for each site has been previously outlined by Saini et al.^39^

**Figure 1:**
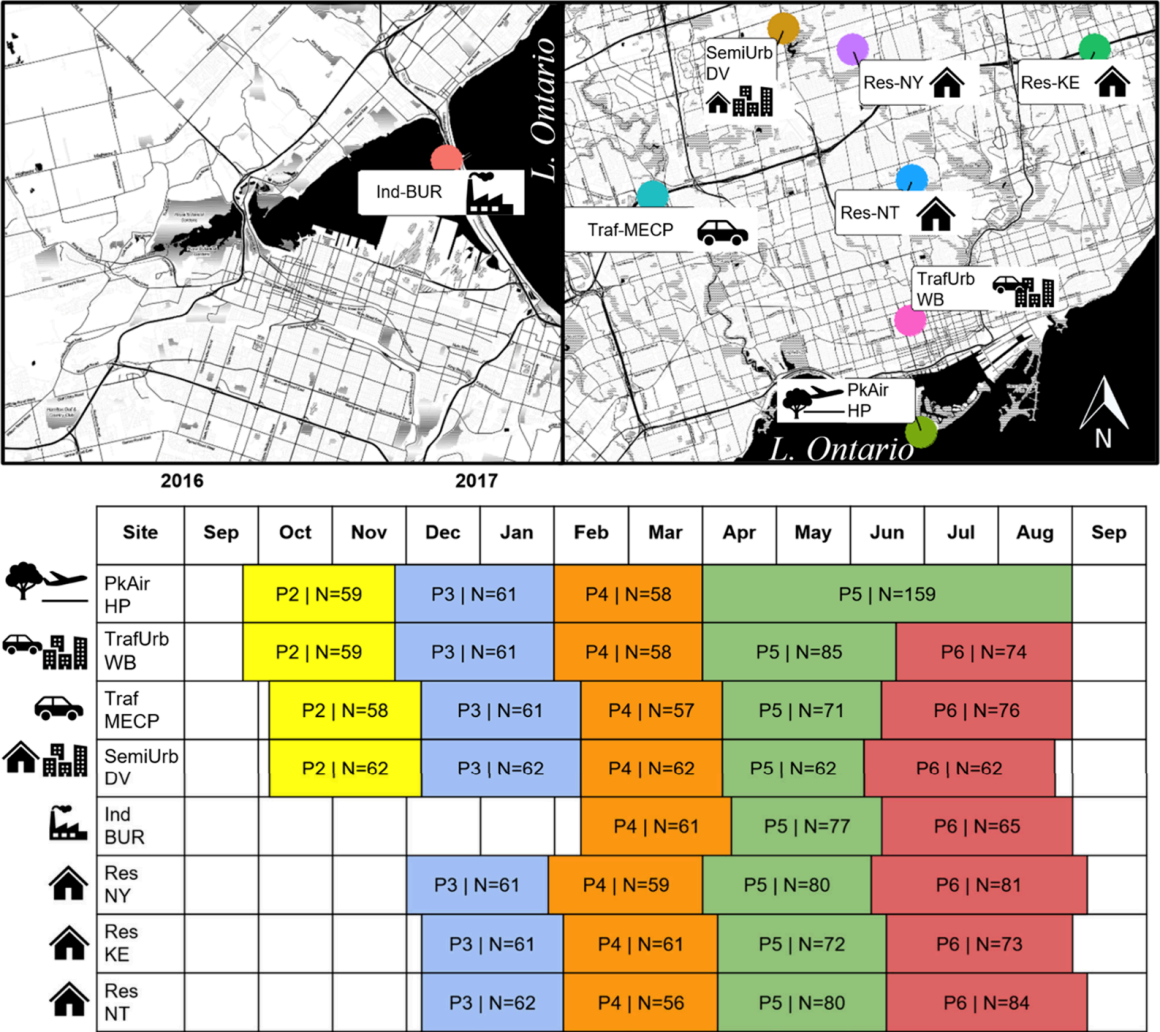
Spatial maps showing location of sampling sites in Burlington (left) and within the Greater Toronto Area (GTA, right). The bottom table shows the sampling periods (P) for which PUF-PAS were collected at each site, where “N” in each box represents the total number of days that the sampler was deployed. Samples P2–6 were included in this study (Oct 2016–Sept 2017) and are numbered for consistency with Saini et al.^39^ Sampler retrieval was not possible at PkAir-HP at the end of Period 5 due to restricted access to the site due to flooding; thus, sampler deployment continued at this location until the end of Period 6. Icons are used to depict source-sector classifications of the sites.

Briefly, PkAir-HP is an island location off the shore of Toronto and consists of lakes and parks, but also has an airport (Billy Bishop Toronto City Airport) 2 km away from the sampling site. The TrafUrb-WB location represents both an urban and traffic source-sector, as it was deployed at the University of Toronto campus in downtown Toronto. Traf-MECP represents a strong traffic source-sector as the sampler was deployed within 20 m of a high-use multilane highway (Highway 401). SemiUrb-DV is a semiurban source-sector, while Ind-BUR is primarily industrial since it is located close to an industrial hotspot in Hamilton Harbour. Finally, Res-NY, Res-KE, and Res-NT all represent residential source types. Figure 1 shows a map of the sampling sites, outlines the sampling periods, and defines their source-sector classification.

The collected PUF disk samples were extracted using ASE 350 with petroleum ether and acetone as solvents (83/17, v/v) at 50 °C and 1500 psi for two cycles (5 min static cycle with 100% flush and 240 s purge).^46^ Sample extracts were concentrated via rotary and nitrogen evaporations, and then subjected to silica column cleanup with Bond Elut SI cartridges (Agilent Technologies Inc.) as described by Rauert et al.^46^ Briefly, the columns were washed with 20 mL of a 50:50 petroleum ether and acetone mixture and then samples were eluted with 40 mL of the same mixture. Eluted extracts were concentrated under nitrogen and reconstituted to 0.5 mL with methanol. Following the analysis for flame retardants, data for which is reported in Saini et al.,^39^ samples were archived and stored at −20 °C and allowed to come to room temperature prior to subsequent analysis. As the sample extracts analyzed in this study are archived samples from a 2016–2017 sampling campaign, the sample extraction procedure was not optimized for the analysis of the target analytes and did not include applicable internal standards for recovery corrections. Subsequent recovery experiments were performed and are described below.

### 2.2 Chemicals

This study initially targeted 17 chemicals associated with tires and vehicle pollution including: DPG, diphenylamine, TMQ, HMMM, benzotriazole, 5-methyl-1H-benzotriazole, benzothiazole, 2-hydroxybenzothiazole, 2-methylbenzothiazole, 2-mercaptobenzothiazole, 2-(methylthio)-benzothiazole, bis(2-naphthyl)-1,4-phenylenediamine (DNPD), N,N′-diphenyl-p-phenylenediamine (DPPD), *N*-isopropyl-N’-phenylenediamine (IPPD), *N*-phenyl-N’-cyclohexyl-p-phenylenediamine (CPPD), 6PPD and its quinone (6PPD-quinone). However, due to low recoveries for some analytes (described later) the remaining qualified data includes 9 chemicals: TMQ, HMMM, 6PPD-quinone, benzotriazole, 5-methyl-1H-benzotriazole, benzothiazole, 2-hydroxybenzothiazole, 2-methylbenzothiazole, and 2-(methylthio)-benzothiazole. The chemical formula and CAS number of each target analyte are provided in Table S1 in the Supporting Information. Table S1 also provides the purchasing information for each analyte, as well as its reported purity. HPLC grade formic acid and methanol were used for calibration curve dilution and mobile phase preparation and were purchased from Fisher Scientific (Ottawa, ON, Canada). Milli-Q water (18.2 MΩ), used for mobile phase production, was produced using a Barnstead system.

### 2.3 Instrumental Analysis

Sample extracts were chromatographically separated using an UltiMate 3000 ultrahigh pressure liquid chromatography system (UPLC) with a Phenomenex (Torrance, CA, USA) Kinetex C18 column (2.6 μm in particle size, 50 × 4.6 mm in length and inner diameter). The injection volume was 25 μL and separation was conducted at room temperature at a flow rate of 500 μL/min. The mobile phase composition and gradient were previously described by Alhelou et al.^47^ A binary gradient was used and consisted of Milli-Q water as phase A and methanol as phase B. Both mobile phases also contained 0.1% formic acid. Over the course of 12.25 min, phase B was increased from 2% to 99% and then held for 2.75 min until starting conditions were re-established in 0.1 min.

The UPLC system was coupled to a Q-Exactive high resolution Orbitrap (Thermo Fisher, Waltham, MA, USA) using heated electrospray ionization. Acquisition was performed using positive ionization under the Parallel Reaction Monitoring (PRM) mode, with the parameters previously described in Johannessen et al.^17^Table S1 outlines the monitored ions, the collision energy, and retention time for each analyte.

### 2.4 Quality Assurance and Quality Control

Field blanks comprising nondeployed PUF-PAS were collected at each site during each period. In addition to the field blanks, laboratory blanks (n = 14) were also processed and analyzed along with the samples. To monitor the instrument performance, a low-concentration quality control (QC) standard was analyzed at the middle of the sample sequence and solvent blanks (methanol) were run frequently.

As previously conducted by Saini et al. for these sample extracts, method detection limits (MDL, pg/m^3^) were estimated by averaging the analyte response in all laboratory and field blanks and dividing the resulting amounts by an air volume of 240 m^3^ (4 m^3^ a day for 60 days) to convert to air concentrations.^39^ The instrument detection limit (IDL) was defined as the amount of target analyte that generates a signal-to-noise ratio of approximately 3:1. The MDLs and IDLs are presented in Table S2.

Recovery experiments were conducted to monitor the extraction efficiency of the method. Five PUF-PAS samples were spiked with 100 ng of each commercial standard and extracted according to the methods reported above. Prior to their analysis on the instrument, each recovery sample was fortified with 10 μL of 6PPD-quinone-D5 (5 ng/μL in methanol) to serve as an injection standard. The fortified samples were then subjected to analysis using the same instrument method as the archived samples.

### 2.5 Data Analysis

Using Genesis integration facilitated by the XcaliburTM software (3.0.63), peak areas were extracted and used for quantification. Calibration curves for each target analyte with a commercial standard were produced in methanol and ranged from 0.006 to 100 ng/mL (R^2^ > 0.98). Where applicable, concentrations were field blank corrected by subtracting the average concentration of the analyte detected in the field blanks at each site.

Instrument concentrations were converted to air concentrations (in pg/m^3^) by dividing the chemical mass in the sample extract by the equivalent air volume (V_eq_) in m^3^. The equivalent air volume was calculated via the following formula:^48^
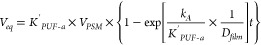
1where K′_PUF-a_ is the unitless PUF-air partition coefficient (K_PUF-AIR_) multiplied by the density of the passive sampling medium (δ_PSM_ in g/m^3^), V_PSM_ is the volume of the passive sampling media (PSM, m^3^), k_A_ is the air-side mass transfer coefficient (m/d), D_film_ is the effective film thickness (m), and t is sampler deployment time (days). For Global Atmospheric Passive Sampling (GAPS)-type shelter PUF-PAS, the δ_PSM_ = 2.10 × 10^4^ g/m^3^, V_PSM_ = 2.10 × 10^–4^ m^3^, k_A_ = 108 m/day, and D_film_ = 5.67 × 10^–3^ m.

K_PUF-AIR_ can be estimated from the octanol-air partition ratio (log K_OA_) from the following equation:^48^
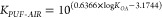
2Thus, to estimate the V_eq_, knowledge of a compound’s log K_OA_ at the average deployment temperature becomes necessary.

The software application COSMOtherm, which implements the COSMO-RS (COnductor-like Screening MOdel for Real Solvents) theory, was selected for calculating these partition ratios. Unlike empirical or group contribution methods, COSMO-RS is a first-principles approach that relies on molecular charge-density distributions derived from density-functional theory (DFT) calculations.^49−51^ This theoretical foundation offers key advantages: it requires no experimental input data, and it employs statistical mechanical treatment of solvent–solute interactions, making it capable of estimating temperature-dependent physicochemical properties over wide temperature ranges.^52^ COSMOtherm was chosen to estimate K_OA_ values due to its demonstrated utility in estimating this property, particularly for volatile compounds with log K_OA_ values below 6.^53^ Here, accurate estimation of the partition ratio for lower log K_OA_ compounds is particularly necessary for determining reliable V_eq_ values, as these compounds may reach equilibrium between the air and the passive sampling medium, rather than remaining in the linear uptake phase throughout the deployment period. Previous evaluations of COSMOtherm estimates have shown root-mean-square errors of 0.3–0.6 log units for Henry’s law constants and K_OA_ values.^52,53^ While this level of uncertainty exists, it is considered acceptable for compounds lacking experimental data.

COSMOtherm directly calculates the air-octanol partition constant according to Henry’s law.^42^ To do so, the structure of each analyte was optimized in COSMOtherm from its SMILES string at the TZVPD-FINE level of theory. The air-octanol partition constant (H_AO_) was then estimated across 19 temperatures ranging from −10 to +30 °C, encompassing the average temperatures experienced by the passive samplers during deployment. H_AO_ was converted to the unitless K_OA_ ratio as outlined by Parnis et al.^52^ Briefly, the K_OA_ values were calculated from H_AO_ using a pure dry octanol molarity of 6.31 mol/L, assuming ideal gas law behavior. Linear regression analysis between the inverse of temperature (1/T, with temperature in K) and the estimated log K_OA_ values provided the necessary A and B terms required for log K_OA_ temperature correction:
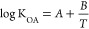
3

A Microsoft Excel template for calculating air volumes and air concentrations from passive air samplers, originally developed by the GAPS Network, was modified to include the temperature-dependent K_OA_ parameters (A and B values) for the target compounds in this study.^41^ This modified template enables standardized calculation of air concentrations for these compounds from passive air sampling data while accounting for temperature effects on sampling rates.

Experimental measurements of the PUF-air partition coefficient, K_PUF-AIR_,^43^ are not yet available for these compounds and would be useful for confirming linear, curvi-linear, or equilibrium phase sampling. Better estimates of K_PUF-AIR_ are especially needed for the chemicals with relatively lower log K_OA_ values such as benzotriazole and benzothiazole, as these compounds are likely to approach equilibrium in the PUF-PAS during deployment, resulting in reduced effective air sample volumes. This effect is enhanced during warmer periods when K_OA_ and K_PUF-AIR_ are lower.

R (v.4.0.5) was used to perform all subsequent data analysis. For statistical analysis, concentrations < MDL were replaced with 1/2 of the compound’s MDLs. Principal component analysis (PCA) was conducted using the *prcomp* function from the stats package (v.3.6.2). The input data set was scaled to have unit variance prior to completing the PCA. This was done to avoid giving too much weight to variables with larger variances. The two principal components that captured the most variation in each analysis were selected and plotted against each other. The *cor* function from the R package *stats* (v.3.6.2) was used with default parameters to calculate Spearman’s rank correlation coefficient (ρ) for each pair of compounds. Correlations with *p* > 0.05 are excluded from the plot.

## 3 Results and Discussion

The results of the recovery experiment are presented in the Supporting Information Table S3. Seven of the target analytes showed low average recoveries (<30%) and were excluded from subsequent data analysis and discussion. Diphenylamine showed elevated average recovery (160%) with high variability (standard deviation = 110 ng) and was also excluded from further consideration. The PPD analytes may have degraded into diphenylamine throughout the recovery experiment to yield these highly variable results. The suboptimal average recoveries observed in this study are consistent with anticipated outcomes when considering the nature of the archived samples and the analytical methods employed. The archived samples were extracted for the analysis of other analytes with different properties and thus was not specifically tuned to capture the analytes of interest in the present study.

This work offers a semiquantitative and preliminary assessment of the occurrence and distribution of selected compounds, many of them established TDCs, within urban air—a domain where current data are notably sparse. However, their actual atmospheric concentrations could potentially be underestimated—by a factor of more than three—due to the low recovery rates observed (e.g., 33% for TMQ and 35% for 2-methylbenzothiazole) (Table S3). Similarly, the concentrations of benzotriazoles reported in our study may be conservatively estimated, potentially undervalued by a factor of approximately two due to recoveries that range from 41 to 61%.

Nevertheless, the comparative data between different samples remain informative. The relative concentrations, even if reported lower than actuality, can still yield insights into the patterns and trends of these compounds’ environmental presence. Therefore, while absolute concentration values must be cautiously considered with an understanding of the probable underestimation, the relative abundance of these analytes across samples offers a meaningful perspective for further research and environmental monitoring endeavors. It is this relative data that can serve as a foundation for subsequent, more refined analytical approaches aimed at quantifying these compounds in urban atmospheres.

### 3.1 Occurrence and Concentrations

**Figure 2:**
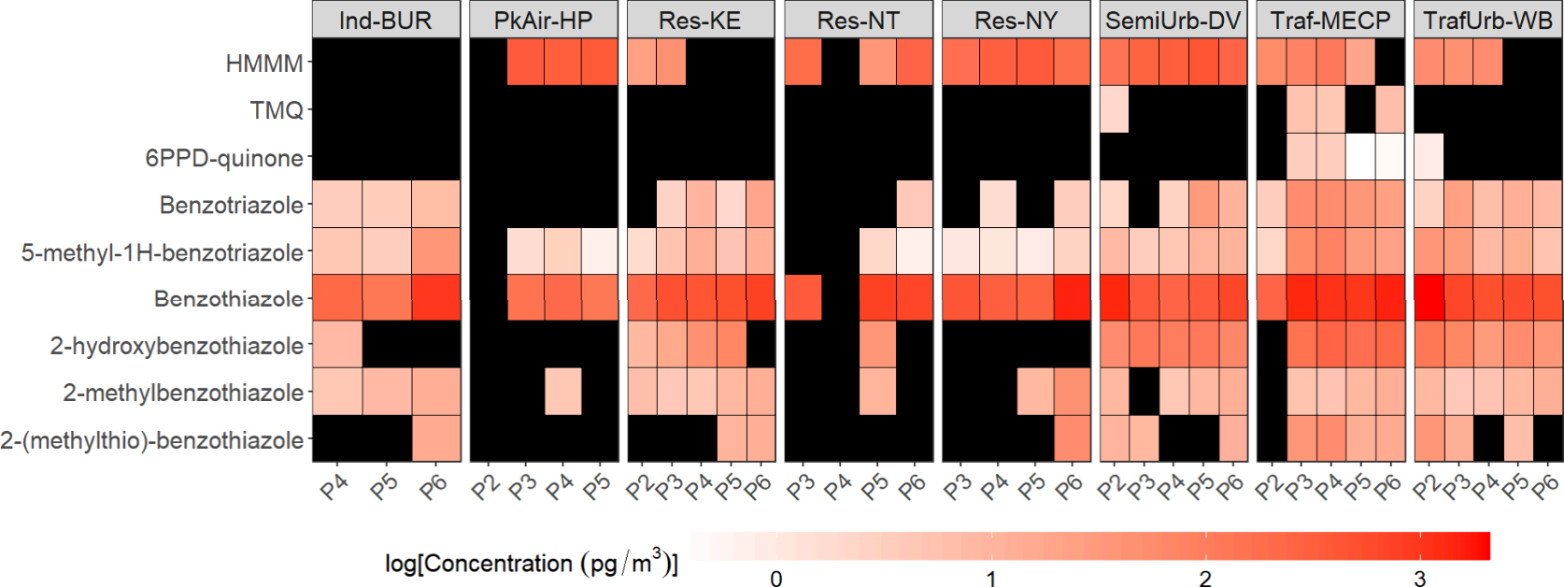
Concentrations in air of tire-derived chemicals from PUF-PAS deployed in the Greater Toronto Area source-sector sites in consecutive sampling periods starting in Oct. 2016 (Period 2, P2) and ending in September 2017 (P6). Black squares represent < MDLs.

Figure 2 shows a heatmap of the contaminant air concentrations from PUF-PAS deployed in the GTA at source-sector resolved sites, obtained in consecutive sampling periods starting in Oct. 2016 (Period 2, P2) and ending in September 2017 (P6). All 9 analytes were detected at concentrations > MDLs (Table S2), with detection frequency in deployed samplers ranging from 11 to 94%, with benzothiazole having the highest detection frequency. Field blank data are reported alongside data from the deployed samples throughout the Supporting Information (Table S4–S6).

HMMM, 2-hydroxybenzothiazole, and benzothiazole, on average, had the highest concentrations within the analyzed samples (Figures 2 and S2, Supporting Information). These data suggest that these compounds have high emissions from their anthropogenic sources into the environment. HMMM also has one of the greatest concentration distributions across analyzed samples (Figure S2). The average HMMM concentration was 190 pg/m^3^, with a maximum concentration of 440 pg/m^3^ detected in spring at the semiurban source-sector (SemiUrb-DV). Our prior analysis of HMMM in PUF-PAS deployed under the GAPS-Megacities initiative frequently detected HMMM in major cities across the globe.^17^ A recent study from the Pearl River Delta in China reported an average and maximum concentration of HMMM of 5.27 pg/m^3^ and 33.3 pg/m^3^, respectively, in PM_2.5_ samples and 0.90 pg/m^3^ and 2.43 pg/m^3^, respectively, in PUF plug gas samples.^18^ There has been no other report of HMMM in air, although it has been frequently detected in urban waterways.^14,22,54−59^ The wide uses and high emissions of HMMM suggested by its frequent detection (69%) and high concentrations (Table S4) make it a chemical of potential environmental concern despite its largely unknown environmental impacts. To further understand the occurrence and fate of HMMM in the urban atmospheric environment, we encourage that additional monitoring studies should include this compound as a target analyte.

Table S4 shows the air concentrations (pg/m^3^) TMQ, which was detected with an average concentration of 4.5 pg/m^3^ (max = 6.0 pg/m^3^). TMQ exhibits potential for considerable developmental toxicity and is likely to pose a significant thyroid hormone disorder risk to a wide range of marine and freshwater aquatic organisms.^60,61^ It has also demonstrated potential carcinogenicity in rats when administered dermally, ingested, and inhaled,^60,62^ which raises concerns regarding the impacts of human exposure. TMQ was previously detected below quantification limits in PUF-PAS deployed in Sydney, Australia.^17^

The concentrations of 6PPD-quinone are included in Table S4. The peak concentration of 6PPD-quinone was recorded at 3.4 pg/m^3^ at Traf-MECP P4, with an average of 1.7 pg/m^3^ (Table S4). This average is higher than the previously reported average for 6PPD-quinone from PUF-PAS deployed in global urban megacities, which was 0.847 pg/m^3^.^17^ However, it is more similar to the highest value reported in the aforementioned global study, which was 1.75 pg/m^3^ in Brazil. Furthermore, the concentrations are higher than those reported for 6PPD-quinone from PUF plug gas samples from the Pearl River Delta (max concentration 0.44 pg/m^3^).^18^ 6PPD-quinone is the transformation product (TP) that readily forms when 6PPD is exposed to ozone.^31^ It is possible that 6PPD is transformed into 6PPD-quinone during the relatively long sample storage to give higher apparent 6PPD-quinone concentrations in the samples, although further QC and quality assurance testing is required under a range of sample treatment and storage conditions (discussed later).

6PPD-quinone has demonstrated pronounced toxicity to a variety of salmonid species,^63−66^ but its toxicity to humans remains unknown. A recent study found 6PPD and 6PPD-quinone to be frequently (60–100%) present in human urine samples.^67^ Concerningly, 6PPD-quinone concentrations (2.91 ng/mL) were the highest in the urine of pregnant women. The presence of these contaminants in the human body and urban air demonstrates the necessity for the toxicity of these compounds to be further investigated.

It should be noted that the PUF-PAS used in this study use the GAPS-type shelter, which has been well characterized against active air samplers. This sampler has been shown to capture both gas-phase and particle-associated contaminants with similar proportions compared to conventional and widely used PS-1 active air samplers.^68^ In addition, Markovic et al. have shown that the GAPS-type housing for the PUF-PAS and the PS-1 type active sampler capture particles up to approximately PM_5_.^45^ As a result, it becomes possible to compare the data generated in this study with active air PM_2.5_ sampling data.

The levels reported in this study for 6PPD-quinone are substantially lower than those detected for 6PPD-quinone in urban PM_2.5_ samples from China from various studies, where maximum concentrations reached 50.5 pg/m^3^ in PM_2.5_ from the Pearl River Delta,^18^ 84 pg/m^3^ in PM_2.5_ from Taiyuan,^20^ 13.8 pg/m^3^ in PM_2.5_ from Hong Kong,^13^ and 7250 pg/m^3^ in PM_2.5_ from Guangzhou.^19^ 6PPD-quinone has also been detected in elevated concentrations in indoor compartments, with an average concentration of 43.0 ng/g in vehicle dust samples.^69^

Although the parent PPDs could not be recovered from the PUF-PAS in the present study, previous work has shown their occurrence in air. 6PPD, IPPD, CPPD, and DPPD have all been detected in air particles, with concentrations up to 6.30 pg/m^3^ in Hong Kong,^13^ in extraction facilitated by ultrasonication of collected air particles with organic solvents. In different studies of PM_2.5_ from China, these compounds were detected in elevated concentrations (sometimes up to the ng/m^3^ range), again facilitated by ultrasonication extraction.^18−20^ These results suggest that future work should integrate the use of organic solvent facilitated ultrasonication in the extraction protocol for PUF-PAS to potentially enhance the recovery of PPD compounds.

The concentrations of benzotriazole and 5-methyl-1H-benzotriazole in air samples from this study are shown in Table S5. The average concentrations of these two compounds were the same when reported to two significant figures: 13 pg/m^3^. Both benzotriazole and 5-methyl-1H-benzotriazole had maximum concentrations at Traf-MECP during P4 (52 pg/m^3^ and 76 pg/m^3^, respectively). Similarly, benzotriazole and 5-methyl-1H-benzotriazole were found to be the dominant benzotriazoles in urban PM_2.5_ from China that were collected in winter.^70^ Benzotriazole and 5-methyl-1H-benzotriazole have demonstrated chronic, acute, and developmental toxicity to an array of aquatic species.^71−73^ Wang et al. suggested that benzotriazole could act as a carcinogen, further emphasizing the significance of its detection in urban air samples.^74^

Table S6 presents the air concentrations for the studied benzothiazoles. Benzothiazole was more frequently detected (94% frequency) than each of its derivatives. Benzothiazole and 2-hydroxybenzothiazole (57% detection frequency) had the highest average concentrations compared to the other benzothiazoles, with average concentrations of 630 pg/m^3^ and 87 pg/m^3^, respectively. Benzothiazole had nearly an order of magnitude higher maximum concentration (2100 pg/m^3^) than 2-hydroxybenzothiazole (240 pg/m^3^), although both concentrations peaked at locations influenced by traffic (TrafUrb-WB and Traf-MECP). Both compounds were detected in higher concentrations here (15× and 14×, respectively) than from passive air samplers that were deployed in global megacities, although the latter were not corrected for equilibrium.^17^

In a recent study from the Pearl River Delta in China, benzothiazole and 2-hydroxybenzothiazole were both detected in elevated concentrations in PM_2.5_ and PUF plug gas phase sampling.^18^ Benzothiazole had an average and maximum concentration of 307 pg/m^3^ and 1200 pg/m^3^, respectively, in PM_2.5_, which is a factor of ∼ 2× lower than the average and maximum concentrations detected herein. However, Tian et al. also detected benzothiazole in average and maximum amounts of 988 pg/m^3^ and 6170 pg/m^3^ in gas phase, which is elevated compared to the current study.^18^ Similarly, the concentrations for 2-hydroxybenzothiazole presented here are much lower than detected by Tian et al.,^18^ where concentrations reached 1130 pg/m^3^ for the particle phase and 298000 pg/m^3^ for the gas phase. Benzothiazole has also been previously detected in concentrations in the ng/m^3^ range from PUF-PAS deployed in end-of-life vehicle dismantling centers, recycling centers, and urban areas in Vietnam.^75^

Among the benzothiazoles studied, 2-methylbenzothiazole showed notable prevalence with a 71% detection frequency, the second highest in the group. However, its concentrations were orders of magnitude (average = 9.4 pg/m^3^, max = 44 pg/m^3^) below those of benzothiazole and 2-hydroxybenzothiazole. These findings can be contextualized against recent measurements from the Pearl River Delta,^18^ where 2-methylbenzothiazole was detected at lower levels in PM_2.5_ (average = 0.49 pg/m^3^, maximum = 4.49 pg/m^3^) but higher concentrations in the gas phase (average = 20.5 pg/m^3^, maximum = 167 pg/m^3^).

2-(methylthio)-benzothiazole was detected less frequently at 40%, with concentrations averaging 22 pg/m^3^ across all sites and peaking at 55 pg/m^3^. These levels differ notably from those reported in the Pearl River Delta,^18^ where the compound showed lower concentrations in PM_2.5_ (average = 2.13 pg/m^3^, maximum = 15.9 pg/m^3^) but substantially higher levels in the gas phase (average = 144 pg/m^3^, maximum = 414 pg/m^3^).

Benzothiazole appears to be acutely toxic to mammals, and its effects are marked by the suppression of both the central nervous system and respiratory functions.^76^ It is also predicted to be acutely and chronically toxic to a range of aquatic organisms.^77^ Both benzothiazole and 2-hydroxybenzothiazole exhibit developmental toxicity and neurotoxicity and induce oxidative stress in juvenile zebrafish.^78^ The human health impacts of benzothiazoles remain a critical, yet largely unexplored, area of study, especially given their reported concentrations in urban air.

### 3.2 Source-Sector Distribution

Figure S3 plots the chemical profiles, sorted by source-sector, of the analytes with air concentrations higher than the MDLs. The analyte that was detected in the second highest concentration (after benzothiazole), HMMM, was present in all investigated source-sectors, excluding the industrial (Ind-BUR) location. HMMM concentrations peaked at SemiUrb-DV, but were elevated in multiple source-sectors including PkAir-HP, Traf-MECP, Res-NT, and Res-NY. The high concentration and high dispersion of HMMM throughout the urban environment is likely attributed to its use as an adhesive and in paints, in addition to its use in tires.^22^ It appears likely that HMMM is emitted from the vehicle paint, top-coats, or tires while stationary, during use, or in car-washes.^79,80^ It may also be continually emitted throughout multiple source-sectors (urban, semiurban, residential) due to its use as a roof coating.^81^ It has also seen utility in antigraffiti coatings as a curing agent, which may be applied throughout the urban landscape.^82^ Tian et al. suggested that HMMM may be a suitable indicator for human activity.^18^

HMMM was also detected in high concentrations (330 – 390 pg/m^3^) at the PkAir-HP location from P3–P5. This is an island and lakeshore site which is surrounded by recreational parkland (Figure S1). However, it is also near (within ∼ 2 km) the Billy Bishop Toronto City Airport and boat and ferry traffic. Thus, elevated concentrations of HMMM at this site may be due to its emissions from the paints/coatings/adhesives used on aircraft and marine vehicles^23−25^ and/or due to its presence in urban plumes coming from the mainland. More research is needed to better understand sources and emissions of HMMM.

TMQ was only detected in the traffic source-sector (Traf-MECP) and once in the semiurban source-sector (SemiUrb-DV), likely due to its use as an antioxidant in tires. 6PPD-quinone was only detected at the traffic-influenced sites, Traf-MECP and TrafUrb-WB (Figure S3). Its sole detection in these source-sectors suggests that proximity to traffic, coupled with the unique air quality conditions driven by traffic, are largely responsible for 6PPD-quinone’s formation and environmental occurrence.

Benzotriazole and its derivative 5-methyl-1H-benzotriazole share similar source-sector chemical profiles (Figure S3). Both compounds exhibited peak concentrations at the Traf-MECP site (max benzotriazole = 52 pg/m^3^, max 5-methyl-1H-benzotriazole = 76 pg/m^3^), which is likely due to their use in automotive components. These compounds also had elevated concentrations at the TrafUrb-WB site (max benzotriazole = 24 pg/m^3^, max 5-methyl-1H-benzotriazole = 39 pg/m^3^). Although both compounds are mainly associated with the traffic source-sector, they also were commonly present at the Res-KE, SemiUrb-DV, and Ind-BUR sites, likely due to their versatile uses as corrosion inhibitors. Both benzotriazole and 5-methyl-1H-benzotriazole are known to be used in aircraft deicers and airfield-pavement deicers, with the methyl derivative often exhibiting higher concentration in these formulations.^83^ The use of 5-methyl-1H-benzotriazole in these products may have resulted in its detection (<MDL – 2.9 pg/m^3^) in the PkAir-HP location. Benzotriazole and 5-methyl-1H-benzothiazole typically had lower concentrations (average benzotriazole = 7.1 pg/m^3^, average 5-methyl-1H-benzotriazole = 6.1 pg/m^3^) in the nontraffic source sectors than those associated with traffic (average benzotriazole = 22 pg/m^3^, average 5-methyl-1H-benzotriazole = 29 pg/m^3^).

Traf-MECP also had the highest concentrations of 2-hydroxybenzothiazole (240 pg/m^3^) and 2-(methylthio)-benzothiazole (55 pg/m^3^). Benzothiazole was present in its highest concentration at the other traffic-influenced site (TrafUrb-WB, max = 2100 pg/m^3^), while 2-hydroxybenzothiazole and 2-(methylthio-benzothiazole) were present here in midrange concentrations (130 pg/m^3^ and 40 pg/m^3^, respectively) compared to their maximum values at Traf-MECP. The elevated concentrations of these benzothiazole derivatives at the traffic source-sectors are expected given the reported levels of these benzothiazoles found in tires by Asheim et al.^36^ Benzothiazole was also present at all other source-sectors in quite elevated concentrations (<MDL – 1500 pg/m^3^, average = 490 pg/m^3^), while 2-hydroxybenzothiazole was also prevalent at SemiUrb-DV and Res-KE, indicating localized emission sources. 2-methylbenzothiazole was detected in all source-sectors with one or two sporadic detections in the Res-NT, Res-NY, and PkAir-HP source-sectors. Its peak concentration was detected in the Res-NY source sector, where 2-(methylthio)-benzothiazole also peaked, although these compounds were both more frequently detected in the traffic associated source-sectors.

**Figure 3:**
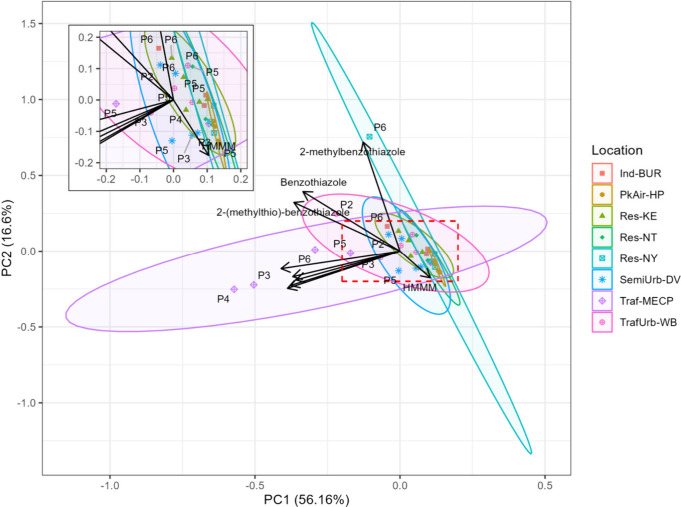
Principal component (PC) analysis on the tire-derived chemicals in source-sector resolved and seasonal data set. PC1 and 2 account for 56% and 17% of the variability in the data set, respectively. Arrows represent loading scores for each compound. Data is categorized by location. Passive air samples were collected from P2 (fall 2016) to P6 (summer 2017) as summarized in Figure 1.

Table S7 shows the summed air concentrations for the benzotriazoles and benzothiazoles groups. The total concentrations of benzotriazoles and benzothiazoles both peaked at traffic influenced sites, namely Traf-MECP in P4 (128 pg/m^3^) for the benzotriazoles and TrafUrb-WB in P2 (2300 pg/m^3^) for the benzothiazoles. This, along with the concentrations for all individual analytes, suggests that the traffic source-sector results in distinct contamination compared to the other investigated source-sectors. Indeed, PCA reveals that the Traf-MECP site has a distinct chemical profile from that of the other locations (Figure 3). The variance of chemical concentrations at the Traf-MECP site can be mostly explained by PC1 and the Traf-MECP site is known to be directly influenced by highway traffic. Consequently, PC1 can be interpreted as traffic influence. As suggested by the loading plot, most compounds analyzed in this study are correlated with PC1 and have traffic origins. Consistently, the data variance at the other site with traffic influence (TrafUrb-WB) can also be mostly attributed to PC1. The chemical profiles of the detected analytes at the Traf-MECP and TrafUrb-WB locations are highlighted in Figure S4. The variances of the chemical concentrations at the other sites are mostly explained by PC2. This suggests that besides direct influence from road traffic, other sources also contribute to some of the analyzed chemicals in urban air. Such sources can be paints, coatings and adhesives in which some of the detected compounds (e.g., HMMM) are known to be used. Nevertheless, these results do suggest that traffic is a predominant source of the analytes in urban air, although there are also a multitude of other anthropogenic sources that deserve further investigation.

### 3.3 Chemical Fate

The PCA (Figure 3) illustrates that the majority of investigated TDCs likely have similar origin and environmental fate to one another, with some key exceptions. HMMM, 2-(methylthio)-benzothiazole, benzothiazole, and 2-methylbenzothiazole are separated from the main cluster of compounds, with higher loadings on PC2 and lower loadings on PC1. These compounds, especially HMMM and 2-methylbenzothiazole, drive PC2, which is likely explained by local nontraffic chemical emissions. Thus, the separation of these compounds from the main cluster suggests that these compounds are not useful as marker chemicals for tire pollution as they likely undergo different emission and fate processes compared to the other analytes.

Figure S6 is the Spearman correlation analysis, illustrating the statistically significant (*p* < 0.05) relationships between investigated compounds. In general, benzotriazoles and benzothiazoles showed strong positive correlations within and between their groups. For example, as indicated by their similar chemical profiles at each site (Figure S3), benzotriazole and 5-methyl-1H-benzotriazole are strongly correlated (Spearman’s ρ = 0.82). 5-methyl-1H-benzotriazole is also strongly correlated to benzothiazole (ρ = 0.70), 2-hydroxybenzothiazole (ρ = 0.73), and 2-(methylthio)-benzothiazole (ρ = 0.71). Benzothiazole is strongly (ρ = 0.80) correlated with 2-(methylthio)-benzothiazole. 6PPD-quinone, which was only detected in the traffic source-sectors, shows a moderate positive correlation with a variety of analytes including TMQ (ρ = 0.67), 2-hydroxybenzothiazole (ρ = 0.63), and 2-(methylthio)-benzothiazole (ρ = 0.63). Future work should continue to monitor the association between these compounds, which are likely to co-occur in urban environments due to their strong traffic emission sources.

To investigate the influence of temperature on the detected levels of the contaminants, correlation analysis was performed on the concentrations (ln *C*) of detected analytes and the inverse of temperatures (1/T, with temperatures in K) at each sampling site (Table S8). The presence of a significant correlation provides insight into emission sources. Here, a significant negative correlation suggests that local volatilization of the chemical during warmer months could be an important source; whereas a significant positive correlation indicates that winter-associated emissions dominate over summer emissions.^39^ Only a few of the target chemicals at only a few of the study sites exhibited significant correlations with temperature. Chemicals with concentrations that increased in air during warmer months included 5-methyl-1H-benzotriazole at SemiUrb-DV site as well as 2-methylbenzothiazole at the Res-KE and the TrafUrb-WB sites, whereas the chemicals that increased in air during colder months included only HMMM at the Traf-MECP site.

### 3.4 Seasonal Differences

HMMM concentrations seem to exhibit seasonal differences such that the compound typically peaks in the spring (e.g., P5 at SemiUrb-DV, PkAir-HP, and Res-NY). At Res-NT, the compound has the highest concentration in summer (P6). HMMM peaks in the winter (P4) at Traf-MECP and has comparable concentrations in both fall (P2) and winter (P4) at TrafUrb-WB. TMQ was detected in comparable concentrations during both the winter (P3 and P4) and summer (P6) at the Traf-MECP site. 6PPD-quinone was detected in highest concentrations at the Traf-MECP site during winter (P4). Previous studies have also noticed a seasonal trend with 6PPD-quinone concentrations increasing in winter.^18,84^

Benzotriazole and 5-methyl-1H-benzotriazole exhibit similar seasonal behavior to each other in each source-sector (Figure S3). For example, at Ind-BUR, they both peak in summer (P6), while they peak in winter (P4) at the traffic site (Traf-MECP). Both 2-hydroxybenzothiazole and 2-(methylthio)-benzothiazole also peak in winter (P4) at the Traf-MECP location, while benzothiazole peaks earlier in the winter during P3 and during summer (P6). Benzothiazole, 2-hydroxybenzothiazole, 2-(methylthio)-benzothiazole, and 5-methyl-1H-benzotriazole seem to exhibit the same seasonal pattern as one another at the TrafUrb-WB site, peaking in fall (P2). Benzothiazole exhibits complex seasonal patterns that vary by source-sector. At TrafUrb-WB site, concentrations peak in fall (P2) before decreasing to moderate and consistent levels. The SemiUrb-DV site shows a similar pattern. By contrast, the Traf-MECP site maintains consistently elevated concentrations throughout winter and summer periods. The Res-KE and Res-NY sites show a different pattern with generally increasing concentrations toward summer. These variable patterns suggest multiple factors influence benzothiazole concentrations beyond simple seasonal trends.

Due to the relatively lower estimated log K_OA_ values for benzothiazoles, they likely approach equilibrium in the PUF-PAS during the typical 2-month deployment period, with faster equilibration during warmer periods resulting in lower sampled effective air volumes. Consequently, their calculated air concentrations are highly sensitive to variations in the temperature-dependent log K_OA_ value, which was estimated via computational methods in this study. Experimental determination of K_PUF-AIR_ (e.g., Francisco et al.; Saini et al.)^43,85^ is needed to further improve the reliability of air concentration calculations for these compounds.

The results of the seasonal behavior investigation indicate that winter brings about unique TDC profiles in air. A box plot showing the distribution of concentrations per sampling period illustrates that the TDCs are more frequently found in higher concentrations in P3 (early winter) compared to the other sampling periods (Figure S5). Furthermore, as previously highlighted, many of the analyzed TDCs typically peak in winter (P3 or P4) in the traffic-associated sites. Gustafsson et al. and Liu et al. showed that the wear of summer/all-season tires was significantly reduced compared to winter tires.^86,87^ Thus, more TWPs seem to be generated from the abrasion of winter tires, which most Ontarians switch to using during the colder months. As larger quantities of TWPs are expected to be generated in the winter, more TDCs are expected to be released into the air. It is also possible that the concentrations of these additives are inherently higher in winter tires than summer, as shown by Asheim et al. for selected benzothiazoles, which could also increase their air concentrations in the winter months.^36^

### 3.5 Guidance for Future Work and TDC Partitioning to PUF Disk and Ambient Particles

Overall, this study demonstrates that although chemicals traditionally associated with tire pollution have many anthropogenic sources, traffic is the primary source of the majority of these chemicals to the atmospheric environment. Elevated concentrations of TDCs in air during colder months and near traffic-impacted locations indicate the importance of traffic-related emissions, particularly in the winter months when the use of more rigid winter tires is believed to increase tire wear. This may have implications for wintertime near-road air quality and inhalation exposure. Future monitoring campaigns should continue to investigate TDCs’ implications for air pollution, especially in regions that experience cold climates.

A key outcome of this work is the addition of partitioning parameters (A and B terms) to the GAPS template, enabling calculation of temperature-specific log K_OA_ values (as well as K_PUF-AIR_) for TDCs, which provides a methodological advance for passive air sampling of these compounds.^41^ The expanded template enables both the prediction of effective air volumes that account for compound-specific sampling behaviors and their temperature dependent uptake profiles, while also providing estimates of gas-particle partitioning using the K_OA_-based model.^44,88^ All 17 TDCs that were initially targeted in this study have been incorporated into the template, providing support for future monitoring campaigns of this structurally diverse class of compounds. Table 1 summarizes the key partitioning parameters and sampling characteristics including the estimated log K_OA_ and K′_PUF-AIR_ of each compound at 25 °C. It also details the equilibrium volume (V_eq_) for 60-day deployments under summer (20 °C) and winter (0 °C) conditions, as well as the particle-phase fractions (ϕ) at these temperatures, as calculated by the template.

**Table 1:** Temperature-Dependent Partitioning Parameters and Sampling Characteristics for Tire-Derived Chemicals (TDCs)^a^

Compound	log K_OA_	K′_PUF-AIR_ (unitless)	Φ, summer	Φ, winter	V_eq_, summer (m^3^)	V_eq_, winter (m^3^)
Benzothiazole	5.85	7.4 × 10^4^	0.00	0.00	20	58
2-Methylbenzothiazole	6.19	1.2 × 10^5^	0.00	0.00	34	96
2,2,4-Trimethyl-1,2-dihydroquinoline (TMQ)	6.83	3.1 × 10^5^	0.00	0.00	83	170
2-(Methylthio)benzothiazole	6.87	3.3 × 10^5^	0.00	0.00	87	173
Benzotriazole	7.63	1.0 × 10^6^	0.00	0.00	165	220
Diphenylamine	7.76	1.2 × 10^6^	0.00	0.01	176	224
5-Methyl-1H-benzotriazole	8.09	2.0 × 10^6^	0.00	0.02	197	230
2-Hydroxybenzothiazole	8.82	5.8 × 10^6^	0.01	0.10	225	237
2-Mercaptobenzothiazole	9.08	8.5 × 10^6^	0.01	0.19	230	238
IPPD	9.95	3.0 × 10^7^	0.10	0.71	237	240
1,3-Diphenylguanidine (DPG)	10.95	1.3 × 10^8^	0.54	0.97	239	240
6PPD	11.14	1.7 × 10^8^	0.65	0.98	240	240
CPPD	11.48	2.9 × 10^8^	0.81	0.99	240	240
DPPD	12.31	9.6 × 10^8^	0.97	1.00	240	240
Hexa(methoxymethyl)melamine (HMMM)	12.79	2.0 × 10^9^	0.99	1.00	240	240
6PPD-quinone	15.07	5.5 × 10^10^	1.00	1.00	240	240
DNPD	16.46	4.2 × 10^11^	1.00	1.00	240	240

a Values include COSMO therm-estimated octanol–air partition ratios (log K_OA_) and PUF–air partition coefficients (K′_PUF-AIR_) at 25 °C, as well as particle-phase fractions (ϕ) and equivalent air volumes (V_eq_) calculated at summer (20 °C) and winter (0 °C) temperatures. The particle-phase fraction was calculated using a total suspended particle (TSP) concentration of 25 μg/m^3^ and an organic matter fraction of particles (f_OM_) of 0.2. The equivalent air volumes were calculated for 60-day deployments. All values were calculated with the Global Atmospheric Passive Sampling (GAPS) template for GAPS-type PUF disk samplers.^41^.

For the more volatile lower K_OA_ compounds like benzothiazole (estimated log K_OA_ = 5.85 at 25 °C), gas-phase sampling dominates and equilibrium between the PUF disk and air is likely reached during typical deployment periods. This is reflected in its relatively low V_eq_ and strong temperature dependence, with summer and winter values of 20 m^3^ and 58 m^3^, respectively. In contrast, compounds with higher log K_OA_ values such as 6PPD-quinone (estimated log K_OA_ = 15.07 at 25 °C) are predicted to be entirely particle-bound (ϕ = 1.00) and maintain linear sampling throughout deployment, achieving the theoretical maximum V_eq_ of 240 m^3^ (4 m^3^/day for 60 days) regardless of temperature. Interestingly, several TDCs exhibit strong shifts in gas-particle partitioning status going from mainly gas-phase in warmer summer months to mainly particle-associated during winter months (Table 2). This has implications for transport, transformation and deposition from air and also for their sampling in air using conventional active and passive air sampling methods.

This novel partitioning information allows for more reliable interpretation of concentration data by accounting for differences between winter and summer deployments, particularly for more volatile compounds that may approach equilibrium in PUF disks during typical deployment periods. The ability to predict both phase distribution and temperature-dependent air sampling volumes provides a stronger foundation for obtaining reliable data and comparing measurements across different seasons.

While these developments strengthen our ability to quantify TDCs in urban air, several methodological challenges remain to be addressed, particularly when using passive PUF disk air samplers. Parent TDCs are inherently reactive, and this reactivity poses obstacles throughout the sampling and analytical process. To improve the methodology for future passive air sampling campaigns, the following guidance should be considered:

1. Evaluate how different conditions, such as atmospheric oxidants, temperature and humidity, affect the stability of TDCs on PUF disks during sampling and after collection (storage prior to extraction). For instance, this could be accomplished by fortifying PUF disk with labeled surrogates of the target analytes prior to their deployment. Oxidation experiments similar to those performed by Jariyasopit et al. would also be insightful and could evaluate the impact of different oxidants and air-sampling substrates.^89^
2. Optimize the PUF disk extraction procedure for parent TDCs and their TPs, which represent a class of compounds with diverse physicochemical properties and evaluate their matrix effects. The extraction of TDCs bound within particles is a particular challenge, as this bound portion may not be directly exchangeable with air.
3. Examine the stability of TDCs in stored extracts, focusing on identifying the most suitable keeper solvents and archival conditions to preserve sample integrity during long-term storage. Storage of PUF disk (with extraction just prior to sample analysis) may be a preferred option, which needs to be evaluated.
4. Calibrate PUF disk samplers for TDCs against field data and confirm which TDCs undergo linear phase sampling through direct, experimental measurements of the K_PUF-AIR_ coefficient (e.g., Saini et al.).^43^ Long-term calibration studies of PUF disk samplers against high-volume air samplers would also be useful in assessing the uptake profile for a wide range of TDCs.
5. Consider the impact of gas-particle partitioning of TDCs on their fate and human/ecosystem exposure pathways in ambient air.

By addressing these challenges, future research can achieve more robust assessments of TDC contamination in urban air, ultimately improving air quality assessments and safeguarding public and ecosystem health.

## Supporting Information Available

Land-use distribution maps (Figure S1); detailed target analyte information (Table S1); detection limits with detection frequencies (Table S2); recovery rates (Table S3); all air concentration data (Tables S4–S7); concentration variability analysis (Figures S2, S5); chemical profiles by source-sector (Figures S3–S4); Spearman correlation matrix (Figure S6); and temperature-dependence analysis (Table S8) (PDF)
