# Supplementary material for: Tire-Derived Organic Chemicals in Urban Air at the Source-Sector Scale and Guidance on the Application of Polyurethane Foam Disk Passive Air Samplers
Source: ACS EST Air. 2025 Mar 28;2(5):917–29. doi: 10.1021/acsestair.5c00013 (PMC12070413; doi:10.1021/acsestair.5c00013)
Supplement: Supplementary file 1 — ea5c00013_si_001.pdf [file ea5c00013_si_001.pdf]

Supporting Information:

Tire-derived organic chemicals in urban air at the source-sector scale and guidance on the application of PUF disk passive air samplers

Cassandra Johannessen[1*], Amandeep Saini[2], Xianming Zhang[1], Tom Harner[2*]

[1] Department of Chemistry and Biochemistry, Concordia University, Montreal, QC, H4B 1R6, Canada

[2]Air Quality Processes Research Section, Environment & Climate Change Canada, Toronto, ON, M3H 5T4, Canada

* Author to whom correspondence should be addressed: E-mail: cassandra.johannessen@mail.concordia.ca or Tom.Harner@ec.gc.ca

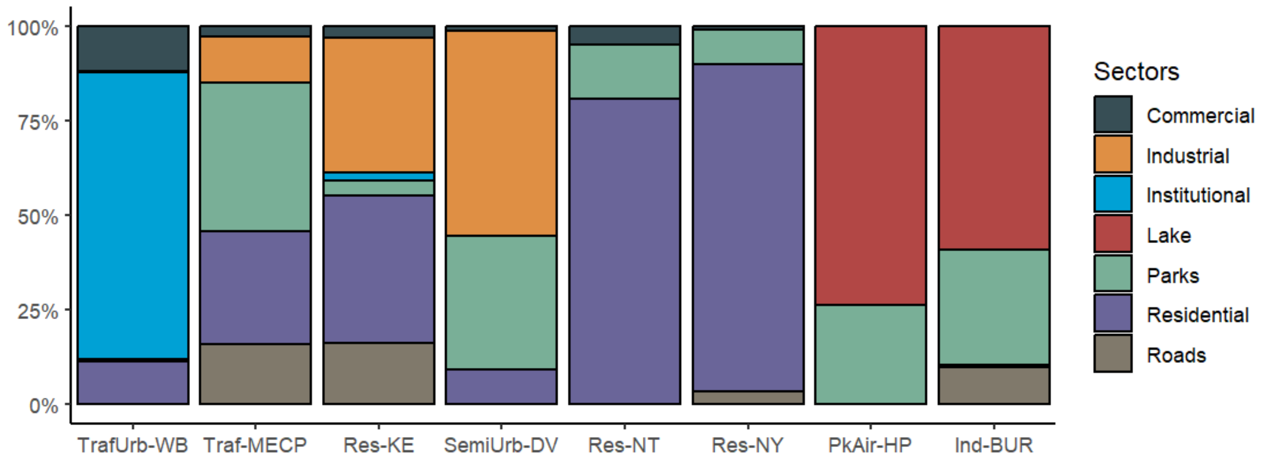

Figure S1: Land-use distribution within 1.5 km radius of sampling sites. Note that this broad-scale land-use characterization may not adequately represent the influence of proximate emission sources. For example, while the Traf-MECP site's 1.5 km land-use profile suggests moderate road influence, its location within tens of meters of Highway 401 (North America's busiest highway) likely makes traffic a significant emission source for this site.

Table S1: Target analytes (name, formula, CAS, purchasing information) included in study. HPLC-HRMS parameters for each analyte are also presented, including the exact mass of the quantifier $[M+H]^+$ and confirmation ion (*m/z*), the fragmentation collision energy (CE), and the retention time (min).

**Compound Name**	**Molecular formula (CAS)**	**Quantifier ion, confirmation ion (*m/z*)**	**Collision energy (CE)**	**Retention time (min)**	**Chemical supplier, purity**
Diphenylguanidine (DPG)	$C_{13}H_{13}N_3$ (102-06-7)	212.1182, 119.0606	30	9.62	Sigma-Aldrich (Oakville, ON), 97%
Benzotriazole	$C_6H_5N_3$ (95-14-7)	120.0556, 65.0387	20	10.28	Sigma-Aldrich (Oakville, ON), 99%
N-isopropyl-N'-phenylenediamine (IPPD)	$C_{15}H_{18}N_2$ (101-72-4)	227.1543, 184.0996	10	11.34	Sigma-Aldrich (Oakville, ON), unreported
5-methyl-1H-benzotriazole	$C_8H_7NS_2$ (136-85-6)	134.0713, 79.0540	20	11.36	Sigma-Aldrich (Oakville, ON), 98%
2-hydroxybenzothiazole	C_7H_5NOS (934-34-9)	152.0165, 124.0213	10	11.99	Sigma-Aldrich (Oakville, ON), 98%
Benzothiazole	C_7H_5NS (95-16-9)	136.0216, 109.1011	10	12.23	Sigma-Aldrich (Oakville, ON), 96%
2-mercaptobenzothiazole	$C_7H_5NS_2$ (149-30-4)	167.9936, 109.0647	30	12.26	Sigma-Aldrich (Oakville, ON), 97%
N-phenyl-N'-cyclohexyl-p-phenylenediamine (CPPD)	$C_{18}H_{22}N_2$ (101-87-1)	267.1856, 185.1071	10	12.49	Toronto Research

					Chemicals (Toronto, ON), >90%
N-(1,3-Dimethylbutyl)-*N′*-phenyl-*p*-phenylenediamine (6PPD)	$C_{18}H_{24}N_2$ (793-24-8)	269.2012, 184.0997	10	12.74	Sigma-Aldrich (Oakville, ON), 95%
Hexa(methoxymethyl)melamine (HMMM)	$C_{15}H_{30}N_6O_6$ (27936-91-0)	391.2300, 283.1510	10	12.97	Toronto Research Chemicals (Toronto, ON), 97%
2-methylbenzothiazole	C_8H_7NS (120-75-2)	150.0372, 109.0105	10	13.16	Sigma-Aldrich (Oakville, ON), 99%
2,2,4-trimethyl-1,2-dihydroquinoline (TMQ)	$C_{12}H_{15}N$ (147-47-7)	174.1277, 127.0346	30	13.46	Toronto Research Chemicals (Toronto, ON), >85%
2-(methylthio)-benzothiazole	$C_8H_7NS_2$ (615-22-5)	182.0093, 166.9855	10	14.12	Sigma-Aldrich (Oakville, ON), 97%
Diphenylamine	$C_{12}H_{11}N$ (122-39-4)	170.0961, 92.0493	30	14.17	Toronto Research Chemicals (Toronto, ON), 96%
N,*N′*-Diphenyl-*p*-phenylenediamine (DPPD)	$C_{18}H_{16}N_2$ (74-31-7)	261.1386, 184.0992	10	14.43	Sigma-Aldrich (Oakville, ON), 98%
6PPD-quinone	$C_{18}H_{22}N_2O_2$ (2754428-18-5)	299.1754, 187.0866	40	14.66	Toronto Research Chemicals

					(Toronto, ON), unreported
6PPD-quinone-D5	$C_{18}H_{17}D_5N_2O_2$ (2750119-14-1)	304.2067	40	14.70	Toronto Research Chemicals (Toronto, ON), 96%
Bis(2-naphthyl)-1,4-phenylenediamine (DNPD)	$C_{26}H_{20}N_2$ (93-46-9)	361.1699, 217.0885	10	15.54	Toronto Research Chemicals (Toronto, ON), unreported

Table S2: Instrument and method detection limits (IDL and MDL) and detection frequency (%) for each target analyte. MDLs (pg/m^3) were estimated by averaging the analyte response in all laboratory and field blanks and dividing the resulting amounts by an air volume of 240 m^3 to convert to air concentrations. The IDL (pg) was defined as the amount of target analyte in a calibration sample that generates a signal to noise ratio of approximately 3:1. Detection frequency calculation does not include field blanks.

**Compound Name**	**Instrument Detection Limit (IDL) (pg)**	**Method Detection Limit (MDL) (pg/m^3)**	**>MDL Frequency (%)**
Hexa(methoxymethyl)melamine (HMMM)	12	5.3	69
2,2,4-trimethyl-1,2-dihydroquinoline (TMQ)	47	0.05	11
6PPD-quinone	2.3	0.10	14
Benzotriazole	47	1.6	69
5-methyl-1H-benzotriazole	12	0.65	91
Benzothiazole	120	15.1	94
2-hydroxybenzothiazole	23	3.1	57
2-methylbenzothiazole	4.7	0.51	71
2-(methylthio)-benzothiazole	47	4.9	40

Table S3: Average recovery and standard deviation of recovery (%) of five spiked PUF-PAS samples.

Analyte	Average Recovery (%)	Standard Deviation (%)
DPG	<MDL	NA
Benzotriazole	41	10
IPPD	<MDL	NA
5-methyl-1H-benzotriazole	47	7.0
2-hydroxybenzotriazole	61	6.9
Benzothiazole	73	14
2-mercaptobenzothiazole	<MDL	NA
6PPD	<MDL	NA
CPPD	<MDL	NA
HMMM	70	19
2-methylbenzothiazole	35	6.8
TMQ	33	3.6
2-(methylthio)-benzothiazole	66	12
Diphenylamine	160	110
DPPD	13	2.5
6PPD-quinone	94	24
DNPD	4.2	1.6

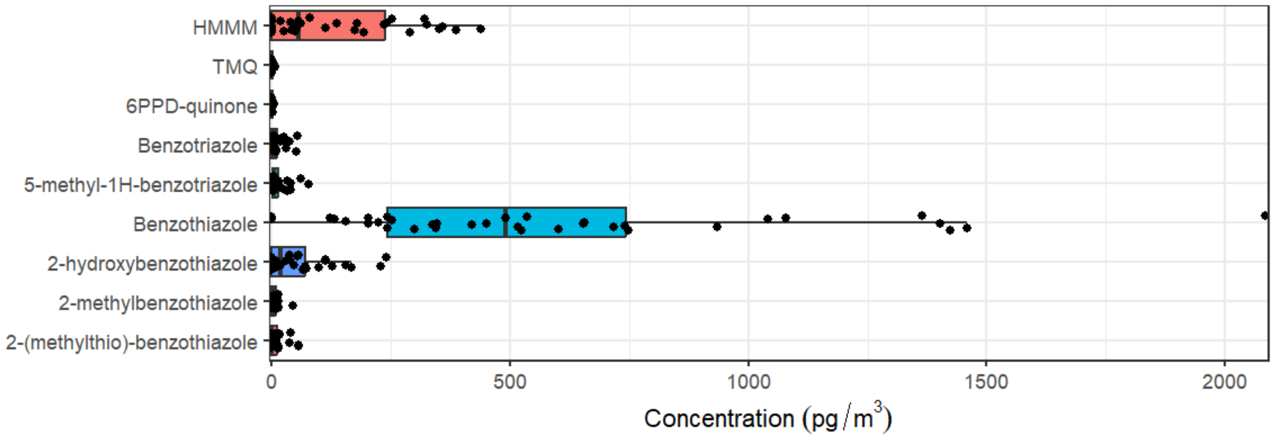

Figure S2: Box plot showing the variability in concentration (pg/m^3) of each detected analyte. Vertical line within box signifies median. Box represents interquartile range (IQR). Maximum whisker span was calculated as 75th Percentile +1.5(IQR). Minimum whisker span was calculated as 25th Percentile -1.5(IQR).

Table S4: Air concentrations (pg/m^3) for HMMM, TMQ, and 6PPD-quinone. <MDL signifies detected below calculated method detected limits. NF signifies that no peak was detected (<IDL). The reported average and range exclude field blank concentrations and only include samples detected >MDL.

	**HMMM (pg/m^3)**	**TMQ (pg/m^3)**	**6PPD-quinone (pg/m^3)**
**TrafUrb-WB FB**	9.3	NF	<MDL
**TrafUrb-WB P2**	55	NF	0.90
**TrafUrb-WB P3**	43	NF	<MDL
**TrafUrb-WB P4**	52	NF	<MDL
**TrafUrb-WB P5**	NF	NF	<MDL
**TrafUrb-WB P6**	NF	NF	<MDL
**Traf-MECP FB**	<MDL	NF	<MDL
**Traf-MECP P2**	61	NF	<MDL
**Traf-MECP P3**	78	5.8	3.2
**Traf-MECP P4**	110	4.3	3.4
**Traf-MECP P5**	19	NF	0.39
**Traf-MECP P6**	NF	6.0	0.47
**Res-KE FB**	7.7	NF	<MDL
**Res-KE P2**	26	NF	<MDL
**Res-KE P3**	48	NF	<MDL
**Res-KE P4**	NF	NF	NF
**Res-KE P5**	NF	NF	<MDL
**Res-KE P6**	NF	NF	<MDL
**Ind-BUR FB**	<MDL	NF	<MDL
**Ind-BUR P4**	NF	NF	<MDL
**Ind-BUR P5**	<MDL	NF	NF
**Ind-BUR P6**	NF	NF	<MDL
**SemiUrb-DV FB**	7.7	0.55	<MDL
**SemiUrb-DV P2**	140	2.1	<MDL
**SemiUrb-DV P3**	250	NF	<MDL
**SemiUrb-DV P4**	320	NF	<MDL
**SemiUrb-DV P5**	440	NF	<MDL
**SemiUrb-DV P6**	240	NF	<MDL
**Res-NT FB**	7.3	NF	<MDL
**Res-NT P3**	180	NF	<MDL
**Res-NT P4**	NF	NF	<MDL
**Res-NT P5**	39	NF	NF
**Res-NT P6**	240	NF	<MDL
**Res-NY FB**	9.3	NF	<MDL
**Res-NY P3**	170	NF	<MDL
**Res-NY P4**	290	NF	<MDL
**Res-NY P5**	360	NF	NF
**Res-NY P6**	190	NF	<MDL
**PkAir-HP FB**	11	NF	NF
**PkAir-HP P2**	NF	NF	<MDL
**PkAir-HP P3**	350	NF	NF
**PkAir-HP P4**	330	NF	<MDL
**PkAir-HP P5**	390	NF	NF
**Average**	190	4.5	1.7
**Range**	19—440	2.1—6.0	0.39—3.4

Table S5: Air concentrations (pg/m^3) for benzotriazole and 5-methyl-1H-benzotriazole. <MDL signifies detected below calculated method detected limits. The reported average and range exclude field blank concentrations and only include samples detected >MDL.

	**Benzotriazole (pg/m^3)**	**5-methyl-1H-benzotriazole (pg/m^3)**
**TrafUrb-WB FB**	<MDL	<MDL
**TrafUrb-WB P2**	2.6	39
**TrafUrb-WB P3**	24	32
**TrafUrb-WB P4**	6.4	8.1
**TrafUrb-WB P5**	13	14
**TrafUrb-WB P6**	7.8	5.1
**Traf-MECP FB**	<MDL	0.82
**Traf-MECP P2**	3.2	2.3
**Traf-MECP P3**	51	61
**Traf-MECP P4**	52	76
**Traf-MECP P5**	37	32
**Traf-MECP P6**	24	23
**Res-KE FB**	<MDL	0.82
**Res-KE P2**	<MDL	1.9
**Res-KE P3**	2.4	5.6
**Res-KE P4**	9.4	13
**Res-KE P5**	2.0	4.9
**Res-KE P6**	18	12
**Ind-BUR FB**	<MDL	0.84
**Ind-BUR P4**	3.3	4.6
**Ind-BUR P5**	3.6	3.5
**Ind-BUR P6**	7.2	38
**SemiUrb-DV FB**	<MDL	<MDL
**SemiUrb-DV P2**	2.4	8.1
**SemiUrb-DV P3**	<MDL	3.3
**SemiUrb-DV P4**	2.8	4.4
**SemiUrb-DV P5**	30.	10.
**SemiUrb-DV P6**	9.8	11
**Res-NT FB**	1.6	0.80
**Res-NT P3**	<MDL	<MDL
**Res-NT P4**	<MDL	<MDL
**Res-NT P5**	<MDL	2.1
**Res-NT P6**	3.9	0.75
**Res-NY FB**	<MDL	<MDL
**Res-NY P3**	<MDL	1.1
**Res-NY P4**	1.6	1.1
**Res-NY P5**	<MDL	0.79
**Res-NY P6**	3.2	2.9
**PkAir-HP FB**	<MDL	<MDL
**PkAir-HP P2**	<MDL	<MDL
**PkAir-HP P3**	<MDL	1.7
**PkAir-HP P4**	<MDL	2.9
**PkAir-HP P5**	<MDL	0.74
**Average**	13	13
**Range**	1.6—52	0.74—76

Table S6: Air concentrations (pg/m^3) for target Benzothiazoles. <MDL signifies detected below calculated method detected limits. NF signifies that no peak was detected (<IDL). The reported average and range exclude field blank concentrations and only include samples detected >MDL.

	**Benzothiazole (pg/m^3)**	**2-hydroxy benzothiazole (pg/m^3)**	**2-methyl benzothiazole (pg/m^3)**	**2-(methylthio)-benzothiazole (pg/m3)**
**TrafUrb-WB FB**	<MDL	<MDL	0.87	<MDL
**TrafUrb-WB P2**	2100	130	9.1	40.
**TrafUrb-WB P3**	720	70.	3.8	13
**TrafUrb-WB P4**	530	30.	4.7	<MDL
**TrafUrb-WB P5**	6.0×10^2	56	8.4	6.2
**TrafUrb-WB P6**	520	36	14	<MDL
**Traf-MECP FB**	<MDL	<MDL	0.84	<MDL
**Traf-MECP P2**	240	<MDL	NF	<MDL
**Traf-MECP P3**	1400	150	5.8	37
**Traf-MECP P4**	1100	240	4.8	55
**Traf-MECP P5**	1.0×10^3	170	9.0	13
**Traf-MECP P6**	1400	230	14	15
**Res-KE FB**	24	<MDL	1.3	6.3
**Res-KE P2**	2.0×10^2	8.3	6.0	<MDL
**Res-KE P3**	490	17	3.9	<MDL
**Res-KE P4**	450	46	4.1	<MDL
**Res-KE P5**	520	71	8.5	11
**Res-KE P6**	750	NF	12	12
**Ind-BUR FB**	21	<MDL	0.59	6.0
**Ind-BUR P4**	220	7.6	4.5	<MDL
**Ind-BUR P5**	130	NF	7.8	<MDL
**Ind-BUR P6**	930	NF	12	15
**SemiUrb-DV FB**	22	6.7	NF	<MDL
**SemiUrb-DV P2**	1400	53	8.5	9.3
**SemiUrb-DV P3**	350	110	NF	8.8
**SemiUrb-DV P4**	240	99	4.3	<MDL
**SemiUrb-DV P5**	350	110	8.8	<MDL
**SemiUrb-DV P6**	650	65	13	11
**Res-NT FB**	27	11	NF	6.4
**Res-NT P3**	340	<MDL	NF	<MDL
**Res-NT P4**	NF	NF	NF	<MDL
**Res-NT P5**	740	36	10.	<MDL
**Res-NT P6**	650	NF	NF	<MDL
**Res-NY FB**	20	5.4	NF	<MDL
**Res-NY P3**	420	<MDL	NF	<MDL
**Res-NY P4**	3.0×10^2	<MDL	NF	<MDL
**Res-NY P5**	250	NF	8.3	<MDL
**Res-NY P6**	1500	NF	44	55
**PkAir-HP FB**	16	<MDL	NF	<MDL
**PkAir-HP P2**	NF	<MDL	NF	<MDL
**PkAir-HP P3**	160	<MDL	NF	<MDL
**PkAir-HP P4**	2.0×10^2	<MDL	4.6	<MDL
**PkAir-HP P5**	120	<MDL	NF	<MDL
**Average**	630	87	9.4	22
**Range**	120—2100	7.6—240	3.8—44	6.2—55

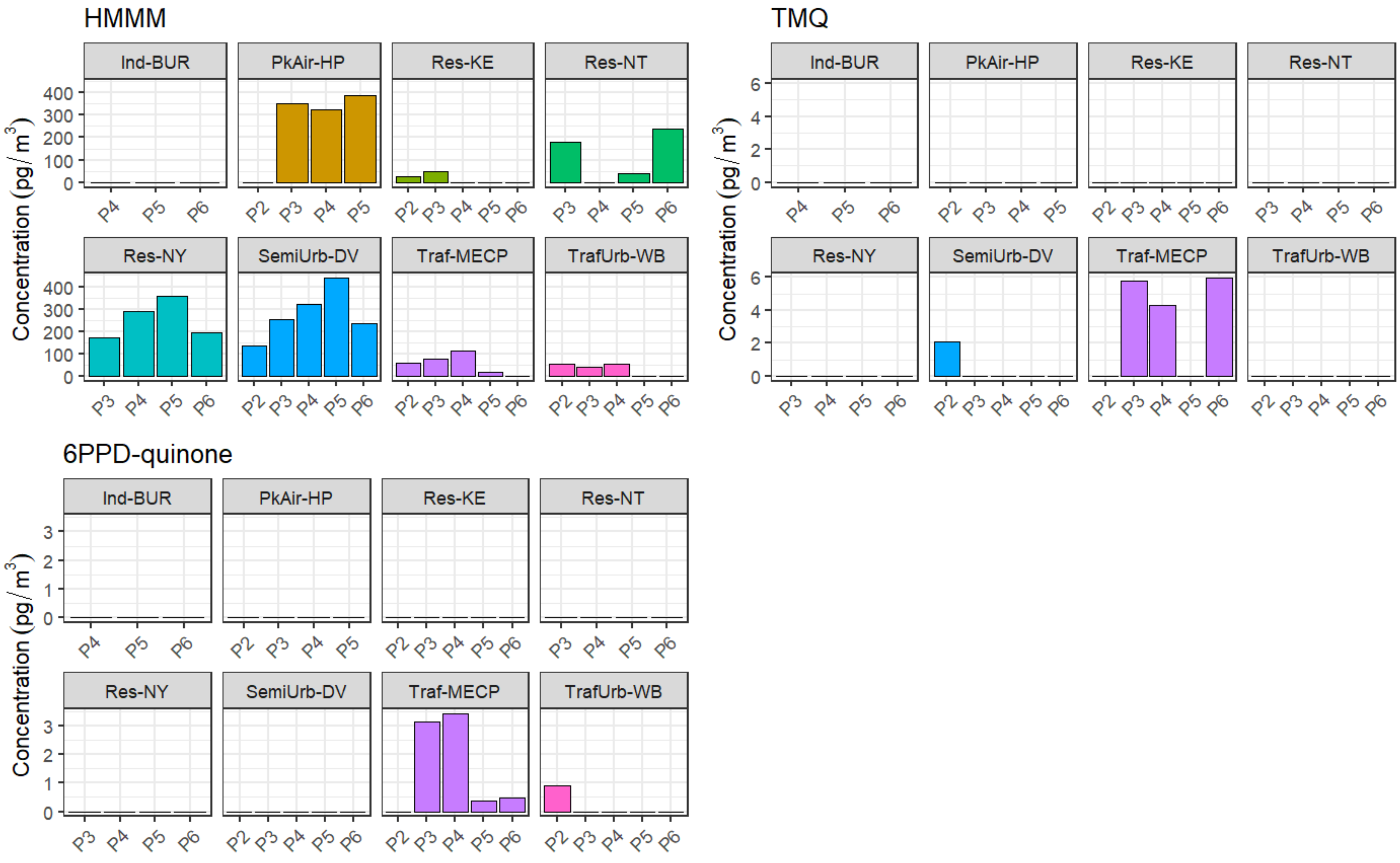
HMMM
TMQ
6PPD-quinone
Concentration (pg/m³)
Ind-BUR
PkAir-HP
Res-KE
Res-NT
Res-NY
SemiUrb-DV
Traf-MECP
TrafUrb-WB
P2
P3
P4
P5
P6

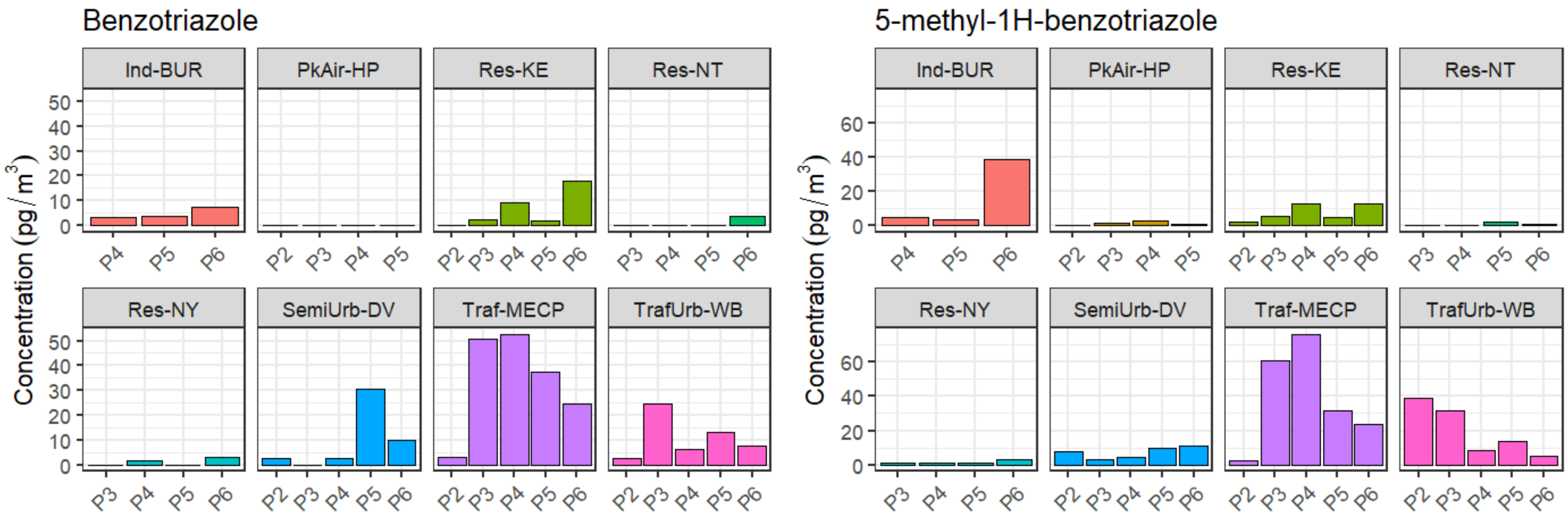
Benzotriazole
5-methyl-1H-benzotriazole
Ind-BUR
PkAir-HP
Res-KE
Res-NT
Res-NY
SemiUrb-DV
Traf-MECP
TrafUrb-WB
Concentration (pg/m3)
P2
P3
P4
P5
P6

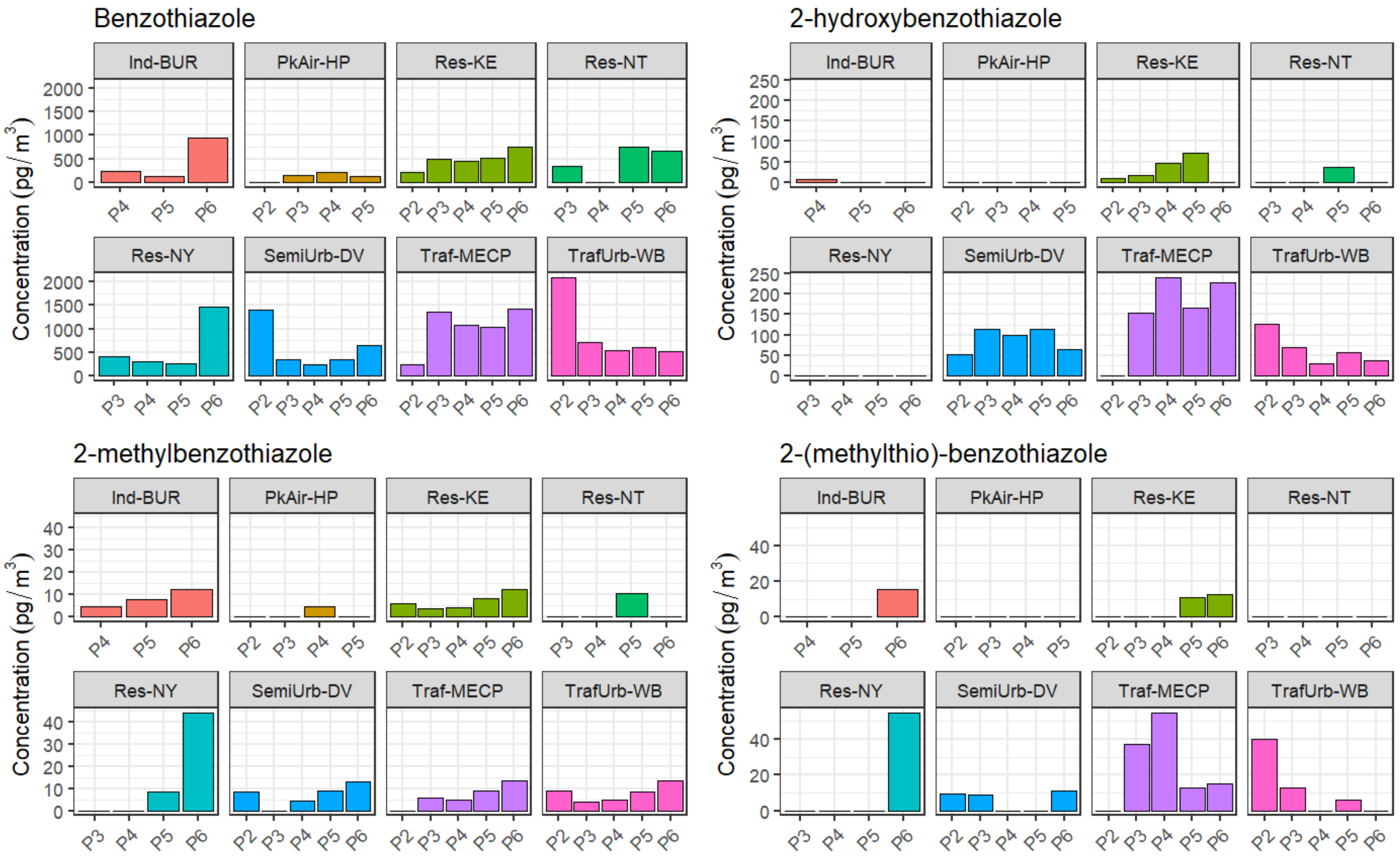

Figure S3: Chemical profiles of select tire-derived chemicals in 8 source-sector resolved sampling sites, as derived from passive air samplers (PUF-PAS) deployed in the Greater Toronto Area in consecutive sampling periods starting in Oct. 2016 (period 2, P2) and ending in September 2017 (P6). The horizontal axis shows the sampling periods per site (P2-P6).

Table S7: Total air concentrations (pg/m^3) summed across analyte group (Benzotriazoles and Benzothiazoles). Field blanks are excluded from summed, average, and range calculations.

	**Total benzotriazole (pg/m^3)**	**Total benzothiazole (pg/m^3)**
**TrafUrb-WB FB**	<MDL	0.87
**TrafUrb-WB P2**	41	2300
**TrafUrb-WB P3**	56	8.0×10^2
**TrafUrb-WB P4**	15	570
**TrafUrb-WB P5**	27	670
**TrafUrb-WB P6**	13	570
**Traf-MECP FB**	0.82	0.84
**Traf-MECP P2**	5.5	240
**Traf-MECP P3**	110	1600
**Traf-MECP P4**	130	1400
**Traf-MECP P5**	69	1200
**Traf-MECP P6**	48	1700
**Res-KE FB**	0.82	32
**Res-KE P2**	1.9	220
**Res-KE P3**	8.0	510
**Res-KE P4**	22	5.0×10^2
**Res-KE P5**	6.9	6.0×10^2
**Res-KE P6**	30.	770
**Ind-BUR FB**	0.84	28
**Ind-BUR P4**	7.8	240
**Ind-BUR P5**	7.1	140
**Ind-BUR P6**	46	960
**SemiUrb-DV FB**	<MDL	28
**SemiUrb-DV P2**	10.	1500
**SemiUrb-DV P3**	3.3	470
**SemiUrb-DV P4**	7.1	350
**SemiUrb-DV P5**	40.	470
**SemiUrb-DV P6**	21	740
**Res-NT FB**	2.4	44
**Res-NT P3**	<MDL	340
**Res-NT P4**	<MDL	<MDL
**Res-NT P5**	2.1	790
**Res-NT P6**	4.6	650
**Res-NY FB**	<MDL	25
**Res-NY P3**	1.1	420
**Res-NY P4**	2.8	3.0×10^2
**Res-NY P5**	0.79	260
**Res-NY P6**	6.0	1600
**PkAir-HP FB**	<MDL	16
**PkAir-HP P2**	<MDL	<MDL
**PkAir-HP P3**	1.7	160
**PkAir-HP P4**	2.9	210
**PkAir-HP P5**	0.74	120
**Summed**	750	23000
**Average**	21	660
**Range**	0.74—130	120—2300

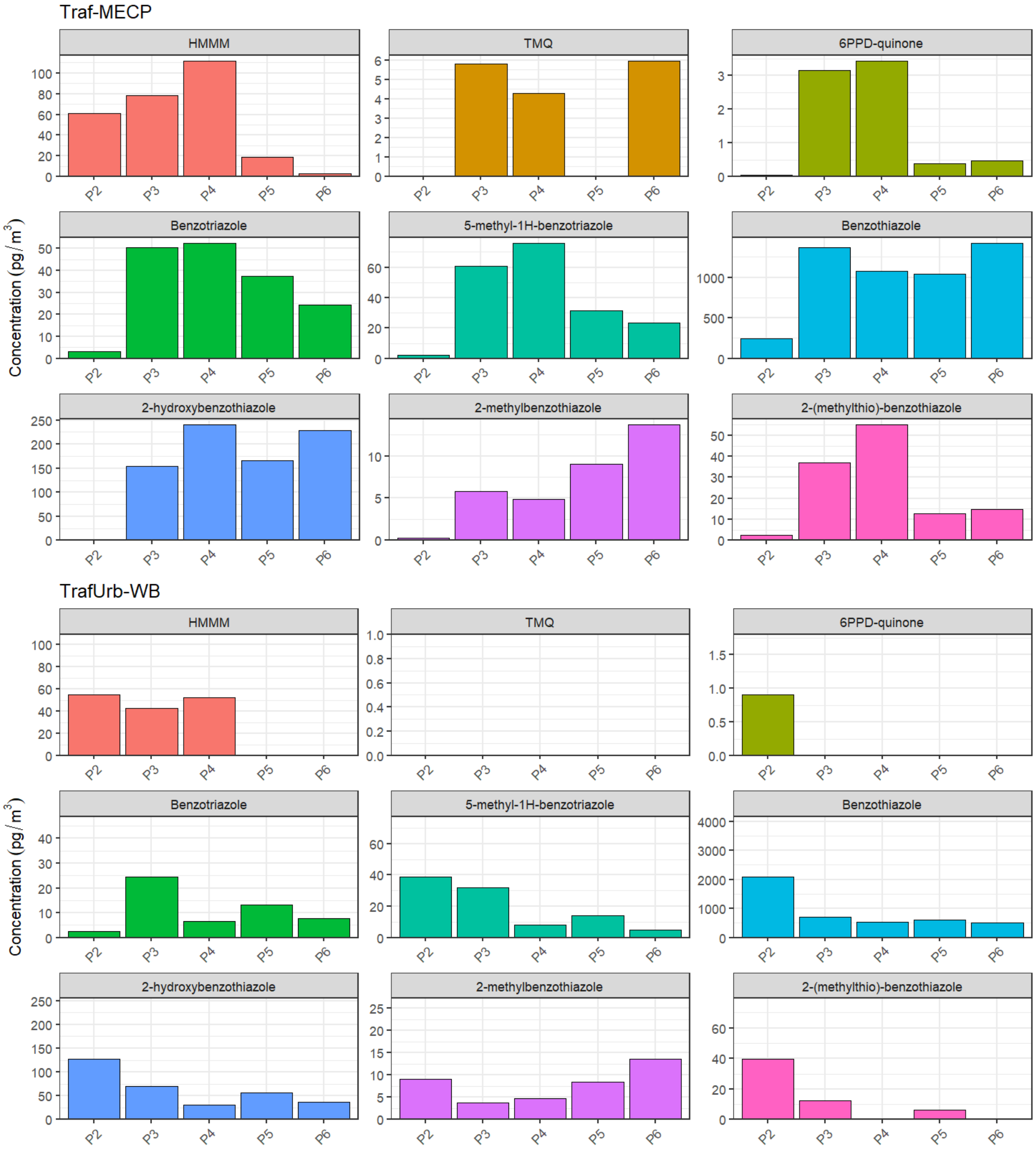

Figure S4: Chemical profiles of detected analytes in the traffic-influenced source-sectors, Traf-MECP and TrafUrb-WB.

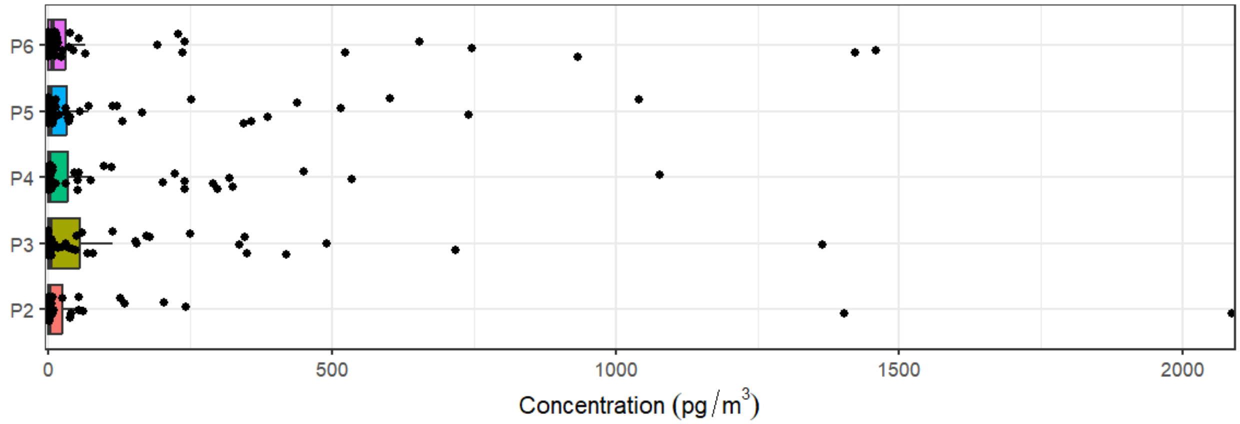

Figure S5: Box plot showing the variability in total analyte concentration (pg/m^3) of each sampled period. Vertical line within box signifies median. Box represents interquartile range (IQR). Maximum whisker span was calculated as 75th Percentile +1.5(IQR). Minimum whisker span was calculated as 25th Percentile -1.5(IQR).

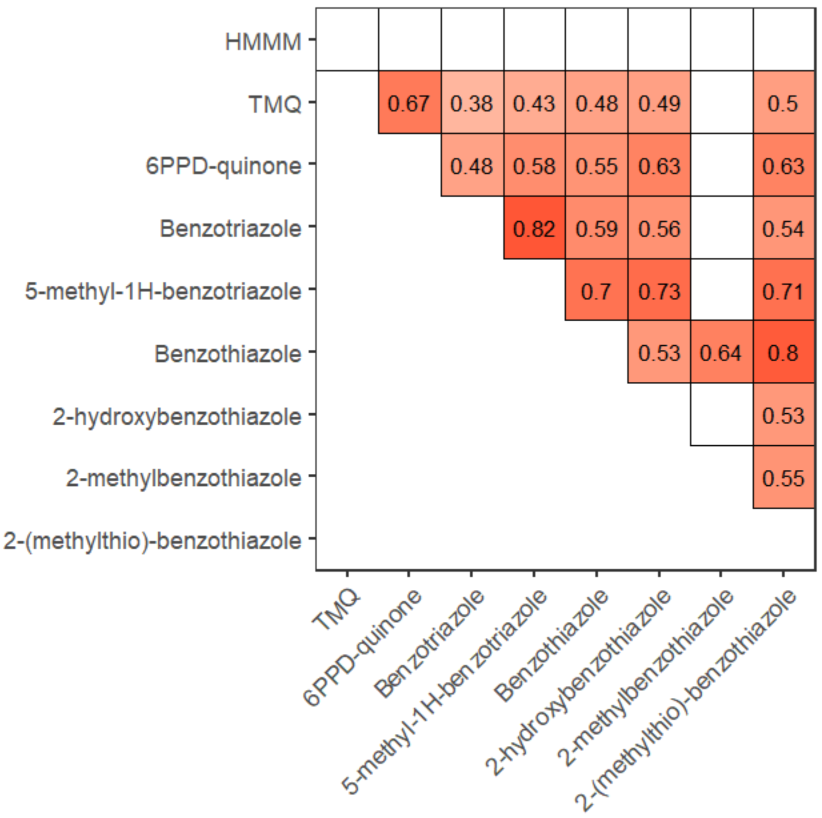

Figure S6*: Spearman correlation* (*ρ*) matrix of the concentration of tire-derived analytes in urban air. Only statistically significant relationships ($p<0.05$) are shown.

Table S8: Correlation (r) obtained by plotting the natural logarithm of concentrations (ln C) of detected analytes against the inverse of temperatures (1/T, Kelvin) at each sampling site. Negative r values (red) denote negative relationship between ln C and 1/T, with higher concentrations in air during warmer months. Only statistically significant relationships are shown ($p < 0.05$). Temperature data was acquired for each sampling site from the closest meteorological stations using the Government of Canada's historical weather database https://climate.weather.gc.ca/climate_data/.

Compound	Pearson's r	Location
2-methylbenzothiazole	-0.99	Res-KE
2-methylbenzothiazole	-0.98	TrafUrb-WB
5-methyl-1H-benzotriazole	-0.96	SemiUrb-DV
HMMM	0.91	Traf-MECP
